# Graphics-processing-unit-accelerated Monte Carlo simulation of polarized light in complex three-dimensional media

**DOI:** 10.1117/1.JBO.27.8.083015

**Published:** 2022-05-09

**Authors:** Shijie Yan, Steven L. Jacques, Jessica C. Ramella-Roman, Qianqian Fang

**Affiliations:** a Northeastern University, Department of Electrical and Computer Engineering, Boston, Massachusetts, United States; b University of Washington, Department of Bioengineering, Seattle, Washington, United States; c Florida International University, Department of Biomedical Engineering, Miami, Florida, United States; d Northeastern University, Department of Bioengineering, Boston, Massachusetts, United States

**Keywords:** Monte Carlo method, light transport, polarization, optical imaging

## Abstract

**Significance:**

Monte Carlo (MC) methods have been applied for studying interactions between polarized light and biological tissues, but most existing MC codes supporting polarization modeling can only simulate homogeneous or multi-layered domains, resulting in approximations when handling realistic tissue structures.

**Aim:**

Over the past decade, the speed of MC simulations has seen dramatic improvement with massively parallel computing techniques. Developing hardware-accelerated MC simulation algorithms that can accurately model polarized light inside three-dimensional (3D) heterogeneous tissues can greatly expand the utility of polarization in biophotonics applications.

**Approach:**

Here, we report a highly efficient polarized MC algorithm capable of modeling arbitrarily complex media defined over a voxelated domain. Each voxel of the domain can be associated with spherical scatters of various radii and densities. The Stokes vector of each simulated photon packet is updated through photon propagation, creating spatially resolved polarization measurements over the detectors or domain surface.

**Results:**

We have implemented this algorithm in our widely disseminated MC simulator, Monte Carlo eXtreme (MCX). It is validated by comparing with a reference central-processing-unit-based simulator in both homogeneous and layered domains, showing excellent agreement and a 931-fold speedup.

**Conclusion:**

The polarization-enabled MCX offers biophotonics community an efficient tool to explore polarized light in bio-tissues, and is freely available at http://mcx.space/.

## 1 Introduction

Polarized light has been found to be highly sensitive to medium structures and hence has been widely adopted in optical imaging to probe microstructural features inside biological tissues.^1^^–^^4^ For example, the polarization status of the backscattered light can be measured to characterize the superficial layer of skin for cancer diagnostic purposes.^5^^,^^6^ The measurements of tissue birefringence permit quantification of abnormalities of the retinal nerve fiber layer^7^ and cornea,^8^ as well as three-dimensional (3D) reconstruction of nerve fiber orientations inside human brains^9^ and orientations of collagen within the uterine cervix.^10^ By measuring the unequal absorption of left-handed and right-handed circularly polarized light, circular dichroism can rapidly determine the folding properties of proteins.^11^ Quantification of collagen and birefringent media alignment can improve evaluation of a therapeutic strategy and its outcome in scar management.^12^ Polarized light imaging (PLI) uses linearly co-polarized images subtracted by those of cross-polarized light to create a differential image based on the small population of superficially scattered photons that still retain much of the incident polarization state.^5^ PLI subtracts the large randomized population of multiple-scattered photons that produce a blinding background of diffuse light. The resulting difference image enhances the contrast of superficial tissue layers and rejects deeper tissue structures, enabling wide-field screening of epidermal or epithelial layers. Mueller matrix polarimetry and the use of various decomposition methods can also be used to pinpoint different regions and structures within biological tissue.^2^^,^^3^ Accurately simulating polarized light transport inside complex tissues allow quantitative investigations of the depth response of polarized light and the perturbations produced by local tissue abnormalities.

The propagation of polarized light inside scattering media can be described by the vector radiative transfer equation (VRTE).^13^ Analytically solving the VRTE is not viable in complex media such as human tissues. Owing to its high flexibility and simplicity in programming, the Monte Carlo (MC) method, among other numerical techniques,^14^^–^^19^ has been one of the limited approaches available to quantitatively model interactions of polarized light with scattering media. Depending on the vectorial representations of polarization states, polarized light MC algorithms can be largely categorized into two formalisms—Jones calculus and Mueller calculus.^2^^,^^4^^,^^20^ The Jones calculus used in the electric-field MC (EMC) algorithm traces the amplitudes and phases of two orthogonal electric-field components (Jones vector) and is therefore well suited for simulating light coherence effects.^21^ On the other hand, the Mueller calculus describes the state of polarization using the Stokes vector.^22^^–^^26^ The Stokes vector does not contain the absolute phase of the electrical field but allows to model unpolarized, partially polarized and fully polarized light. It can be obtained by measuring four intensity values. Similarly, Mueller matrices can be obtained through 16 intensity measurements and using Mueller matrix decomposition, quantities, such as tissue retardation, depolarization and de-attenuation can be obtained.^4^

A well-known limitation of MC methods is the long computation time. Due to the rapid emergence of massively parallel computing techniques, benefit largely from the fast advances in many core processors such as graphics processing units (GPUs), MC simulations of polarized light have seen significant speed improvement over the past decade. Several groups have reported parallel EMC implementations.^27^^–^^29^ Wang et al.^27^ presented a compute unified device architecture (CUDA)^30^-based EMC to model coherent light in a single homogeneous slab and achieved over 370× speedup compared to the central processing unit (CPU) counterpart.^21^ Ding et al.^29^ extended the GPU-based EMC algorithm to consider multi-layered media at the expense of reduced speedup (∼45×). In addition, Li et al.^31^ presented a CUDA-based polarized light MC algorithm to model interstitial media embedded with spherical and cylindrical scatterers. It employed a single-kernel scheme and was hundreds of times faster than its CPU version.^26^ In 2019, Oulhaj et al.^32^ reported a GPU-accelerated MC algorithm to efficiently compute the sensitivity profile for polarized light inside homogeneous media. The reported GPU implementation was verified against a widely used CPU-based code developed by Ramella-Roman et al.^24^ and reported over 150× speedup.

Although these studies have demonstrated significantly improved simulation speed, most of these simulators only support layered domains and can not address the needs in modeling increasingly complex media.^4^ In the simulation of biological tissues with irregular-shaped structures, employing simplifications in domain geometries could introduce significant errors. For example, in the MC modeling of human brains, noticeable differences have been observed between layered-slab models and more anatomically realistic models such as voxel-based and mesh-based brain models.^33^

In this work, we present an open-source and GPU-accelerated MC simulator to model polarized light inside 3D heterogeneous media. This MC algorithm utilizes a 3D voxelated grid to represent spatially varying distributions of spherical scatterers, characterized by their radii and densities. We use Muller calculus to update the Stokes vectors of simulated photon packets, from which we can compute various polarimetry related measurements along the surface of the domain. In the remainder of this paper, we first briefly review the steps of the meridian-plane MC algorithm.^24^ Then we detail our GPU-implementation of this algorithm, as part of our enhanced open-source MC software—Monte Carlo eXtreme (MCX),^34^ including the preprocessing steps to encode the distribution of particles into a 3D array data structure and optimization strategies to better use GPU resources. In Sec. 3, we validate the proposed GPU-based polarization-enabled MCX (pMCX) against the widely used CPU MC simulator “meridianMC” written by Ramella-Roman et al.^35^ and quantify the speed improvement using several benchmarks of homogeneous and heterogeneous domains. Finally, we summarize the key findings and discuss future directions.

## 2 Methods

### 2.1 Meridian-Plane Polarized Light MC

The meridian-plane polarized light MC algorithm^35^ largely follows the standard MC photon transport simulation steps,^36^ including “launch,” “move,” “absorb,” “scatter,” and “detection.” At the “launch” stage, the initial weight, position, and direction vector are defined for each photon packet depending on the profile of the incident beam. To describe the polarization state, the Stokes vector is defined with respect to the initial meridian plane for every simulated photon packet.^24^ The meridian plane is defined by the plane spanned by the photon propagation direction and the z axis, as shown in Fig. 2 in Ref. 24. The Stokes vector $\overset{\to }{S}$ consists of four quantities [I,Q,U,V], where I (I≥0) describes the total light intensity, Q (−1≤Q≤1) controls the mixing between horizontally (Q=1) and vertically (Q=−1) linearly polarized light, U (−1≤U≤1) controls the mixing between +45  deg (U=1) and −45 deg (U=−1) linearly polarized light, and V (−1≤V≤1) controls the mixing between right (V=1) and left (V=−1) circularly polarized light.^1^

After the “launch” step, the photon packet starts propagating inside the simulation domain. In lossy media, the packet weight is monotonically reduced along the photon’s paths and the weight loss is accumulated into the local grid element (such as a voxel or tetrahedral element^37^). When arriving at an interaction site, the photon packet changes direction due to scattering. To compute the new direction cosines, the scattering zenith angle θ (0≤θ≤π) and azimuth angle ϕ (0≤ϕ<2π) are statistically sampled. Compared to the standard MC, the scattering step in a polarized light simulation requires additional computation to properly update $\overset{\to }{S}$. First, the probability density function (also known as the scattering phase function) of polarized light has a bivariate dependence on both θ and ϕ. For incident light with a Stokes vector ${\overset{\to }{S}}_{in}=[I_{in},\;Q_{in},\;U_{in},\;V_{in}]$, the phase function P(θ, ϕ)^24^ is

$$P(\theta ,\phi )=s_{11}(\theta )I_{in}+s_{12}(\theta )[Q_{in}\cos(2\phi )+U_{in}\sin(2\phi )],$$ (1)

where $s_{11}(\theta )$ and $s_{12}(\theta )$ are elements from the scattering matrix M(θ) from a homogeneous spherical particle, computed via the Mie theory^38^

$$M(\theta )=[\begin{array}{cccc} s_{11}(\theta ) & s_{12}(\theta ) & 0 & 0\\ s_{12}(\theta ) & s_{11}(\theta ) & 0 & 0\\ 0 & 0 & s_{33}(\theta ) & s_{34}(\theta )\\ 0 & 0 & -s_{34}(\theta ) & s_{33}(\theta ) \end{array}].$$ (2)

A rejection method^24^ is employed to select both angles θ and ϕ. Once θ and ϕ are determined, the Stokes vector must be rotated relative to the new meridian plane using M(θ) to update the polarization states.

To efficiently perform the rejection method and Stokes vector rotation, the elements of the scattering matrix M(θ) of all user-specified spherical scatter species are pre-computed over a discreteized set of θ. A photon packet is terminated when it escapes from the simulation domain or, if enabled, fails to survive a Russian roulette.^36^ It is noteworthy that the original meridian-plane polarized light MC assumes refractive-index matched domain boundaries.^24^ The Stokes vector of the escaping photon is rotated relative to the meridian plane of the detector placed immediately outside the domain boundaries, before being accumulated to generate desired output quantities. A detailed description of the formulas used in the meridian plane MC algorithm can be found in the literature.^24^

### 2.2 Implementing Meridian Plane MC in MCX

**Fig. 1:**
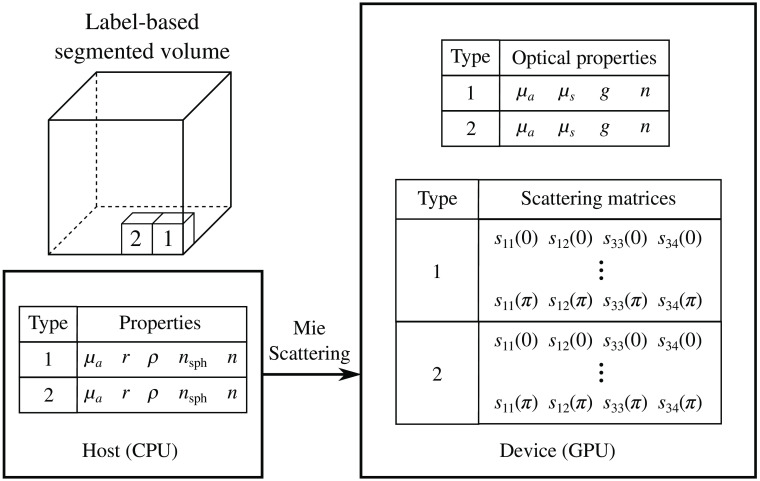
Media representation and media data preprocessing in a polarization-enabled Monte Carlo simulation.

The original CPU-based meridian-plane MC program,^35^ referred to as “mcMeridian” (stok1.c) hereinafter, is dedicated to modeling homogeneous infinite slab geometries. In contrast, the CUDA-based MCX is capable of modeling arbitrarily heterogeneous media represented by a 3D voxelated domain.^34^ In a non-polarization MCX simulation, the domain is represented by a 3D integer array with each number representing the index or label of the tissue at each voxel. The actual optical properties of the tissue label are stored in a look-up table, with four element per tissue type: absorption coefficient $\mu_{a}$ (1/mm), scattering coefficient $\mu_{s}$ (1/mm), anisotropy g, and refractive index n. To simulate polarized light, the scattering properties of each type of scatterer must be included in addition. To simply the computation, here we only consider spherical scatterers. The radius r ( μm), refractive index $n_{\mathrm{sph}}$, and volumetric number density ρ ($1/\;\mu m^{3}$) of the spherical particle scatters can be specified for each tissue type. When spherical particle properties are specified, the corresponding $\mu_{s}$, g, and elements of the scattering matrix M(θ) are pre-computed using the Mie theory^39^ on the host (i.e., CPU). As shown in Eq. (2), the scattering matrix of homogeneous spherical scatterers consists of four independent floating-point numbers $s_{11}(\theta ),s_{12}(\theta ),s_{33}(\theta )$, and $s_{34}(\theta )$. In our implementation, the scattering parameters are sampled at 1000 evenly spaced points between 0 and π, as done in mcMeridian.^35^ The pre-computed optical properties and scattering matrix data are then transferred to the device (i.e., GPU), as shown in Fig. 1.

## 3 Results and Discussions

In this section, we first validate the aforementioned pMCX in a homogeneous slab using the single-threaded CPU-based implementation (mcMeridian^24^) as a reference, which has been extensively used by the community and validated by experimental studies.^25^ The speed improvement is also quantified. It is noteworthy that mcMeridian^35^ simulates an infinite slab media geometry in the x/y directions, whereas in an MCX simulation, a photon is confined inside a bounding box with user-specified dimensions.^34^ To ensure that our speed comparison is valid, we modified the source code of mcMeridian and added an implicit bounding box to match the dimension settings in pMCX. In the first benchmark, the simulation domain is a $20\times 20\times 10\;{\mathrm{mm}}^{3}$ homogeneous slab, the Mie scattering parameters of the embedded spherical scatterer are $\mu_{a}=0\;{\mathrm{mm}}^{-1}$, r=1.015  μm, $\rho =1.152\times 10^{-4}\;\mu m^{-3}$, and $n_{\mathrm{sph}}=1.59$. The refractive index of the background medium is n=1.33. A monochromatic pencil beam source is positioned at the bottom center (10,10,0)  mm of the domain, pointing toward the +z axis and emitting horizontally polarized light at wavelength λ=632.8 nm. The initial Stokes vector of the incident beam is $\overset{\to }{S}=[1,1,0,0]$. The backscattered photons are collected by a square-shaped area detector ($20\times 20\;{\mathrm{mm}}^{2}$) placed on the boundary at z=0  mm. In this benchmark, $10^{8}$ photon packets are simulated on a desktop running Ubuntu 18.04 with an Intel i7-6700K CPU and an NVIDIA RTX 2080 GPU.

**Fig. 2:**
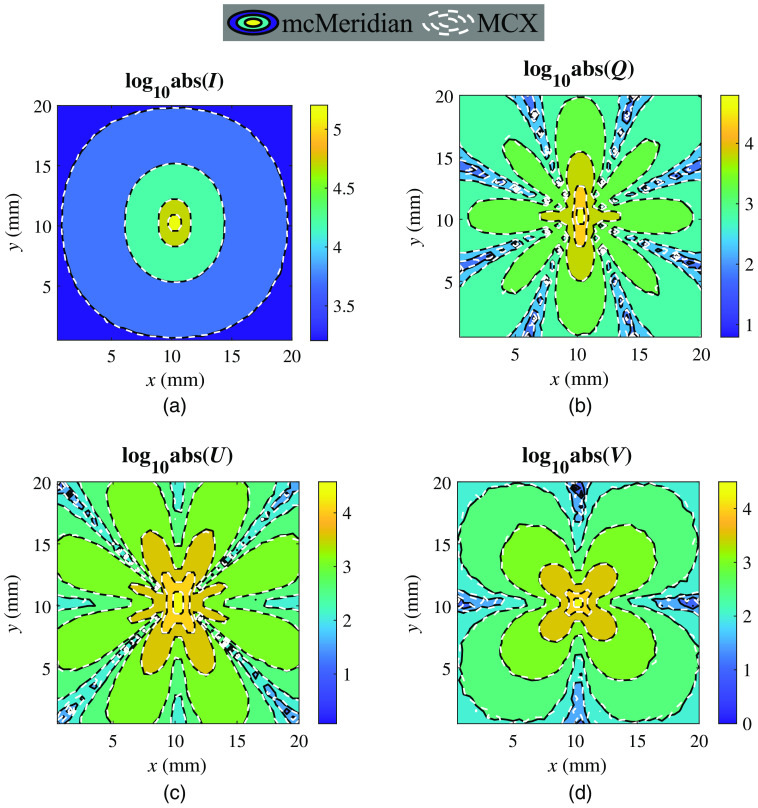
Contour plots of the absolute backscattered (a) I, (b) Q, (c) U and (d) V (in $\log_{10}$ scale) generated by mcMeridian (black solid lines) and pMCX (white dashed lines).

In Fig. 2, we compare the distribution of backscattered [I,Q,U,V] components using contour plots in MATLAB (MathWorks, Inc., Natick, Massachusetts, United States) and observed excellent agreement between mcMeridian and pMCX solutions. For further quantitative analysis, the root-mean-square errors of I,Q,U,V (in $\log_{10}$ scale) between mcMeridian and pMCX are computed on the matching detector area ($20\times 20\;{\mathrm{mm}}^{2}$), reporting 0.0076, 0.0881, 0.1015, and 0.0938, respectively. We measure the total runtimes, including input data preprocessing, photon transport simulation, and output image generation, with mcMeridian reporting 18111.44 s using the Intel CPU and pMCX reporting 19.45 s on the NVIDIA RTX 2080 GPU, suggesting a 931× speedup. In addition, we also benchmark simulation speeds when storing the scattering matrix data over different GPU memory locations, including global, shared, and constant memories.^30^ The global memory implementation reports the fastest speed at 8401 photons/ms, followed by the shared memory (4965 photons/ms) and constant memory (2182 photons/ms) implementations. Although the shared memory is known to be the fastest among the three memory types, its has a very small size, up to 48 KB per block.^30^ For storing the scattering matrix of a single species of scatterer at 1000 angular steps, a total of 16 KB memory is needed. Allocating a large amount of shared memory can lead to drastically reduced active block number, which explains the lower speed compared to the global memory case. On the other hand, constant memory also has a small size (64 KB).^30^ It is most efficient when a memory value is being reused many times after a single read. However, the use of the rejection method requires random access to the buffer which fails to be accelerated by the constant memory due to high “cache-miss.”^30^

In the next benchmark, we further validate our pMCX simulator by comparing with an extended mcMeridian (with added support of layered media) in a two-layer domain. The slab-shaped simulation domain has a size of $100\times 100\times 50\;{\mathrm{mm}}^{3}$ with the thickness of the superficial layer, $d_{e}$, ranging between 0 and 10 mm. The Mie scattering parameters are λ=632.8  nm, $\mu_{a}=0.001\;{\mathrm{mm}}^{-1}$, sphere radius r=0.05  μm, number density $\rho =19.11\;\mu m^{-3}$, $n_{\mathrm{sph}}=1.59$, and n=1.33 for the superficial layer. The bottom layer has r=0.3  μm, $\rho =2.198\times 10^{-2}\;\mu m^{-3}$, and the other parameters are the same as the superficial layer. These choices of r and ρ yield a reduced scattering coefficient $\mu_{s}^{\prime }=\mu_{s}\cdot (1-g)=1\;{\mathrm{mm}}^{-1}$ for both layers, along with the same absorption $\mu_{a}$ and hence approximately the same reflected intensity I for all $d_{e}$. A pencil beam is located on the surface of the slab at (50,50,0)  mm, pointing toward the +z axis and emitting horizontally polarized light, with the initial Stokes vector $\overset{\to }{S}=[1,1,0,0]$. A total of $10^{7}$ photon packets are simulated for both mcMeridian and pMCX.

**Fig. 3:**
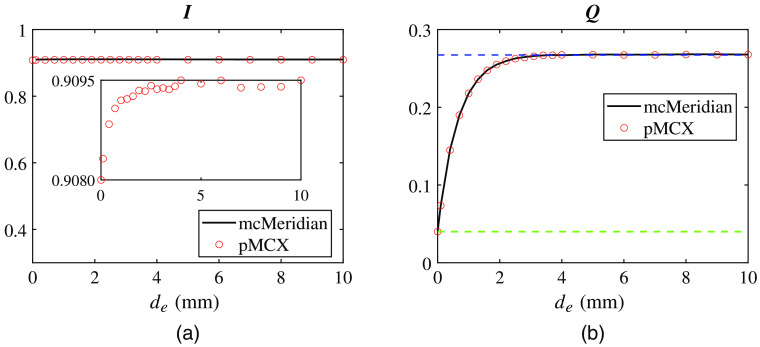
Validation of pMCX in a two-layer domain. We plot the backscattered (a) I and (b) Q components computed by pMCX and mcMeridian as the superficial layer thickness ($d_{e}$) increases from 0 to 10 mm. Two dashed lines in (b) indicate back-scattered Q values computed from a homogeneous slab filled only with the medium of the bottom layer (green) and that of the superficial layer (blue). The inset in (a) shows a zoom-in view of the y axis of the I component obtained by pMCX to demonstrate subtle variations due to sub-diffusive scattering effect.

In Fig. 3, we plot the total reflected I and Q components as a function of the superficial layer thickness $d_{e}$. We do not include total U and V plots because they are nearly zeros across all $d_{e}$ values, this is expected as U and V distributions sum to zeros due to symmetric positive and negative components. The outputs from mcMeridian (black solid lines) and those from pMCX (red circles) once again show excellent agreements. The plot of Q increases with the thickness $d_{e}$ of the superficial layer, which contains smaller spherical scatters and hence stronger back-scattering than the deeper layer. With thickness $d_{e}$ growing from 0 to 1 cm, the total I value shows a minuscule increase by 0.17% (from 0.9080 to 0.9095), as shown in the inset in Fig. 3(a), as a result of sub-diffusive scattering. The two-phase transition of Q matches our expectations: when the superficial layer is very thin, the reflectance values are close to the value as if the domain is entirely filled with the bottom medium (green dashed line); as we increase $d_{e}$, the reflectance values asymptotically approach those determined by the media in the superficial layer (blue dashed line).

**Fig. 4:**
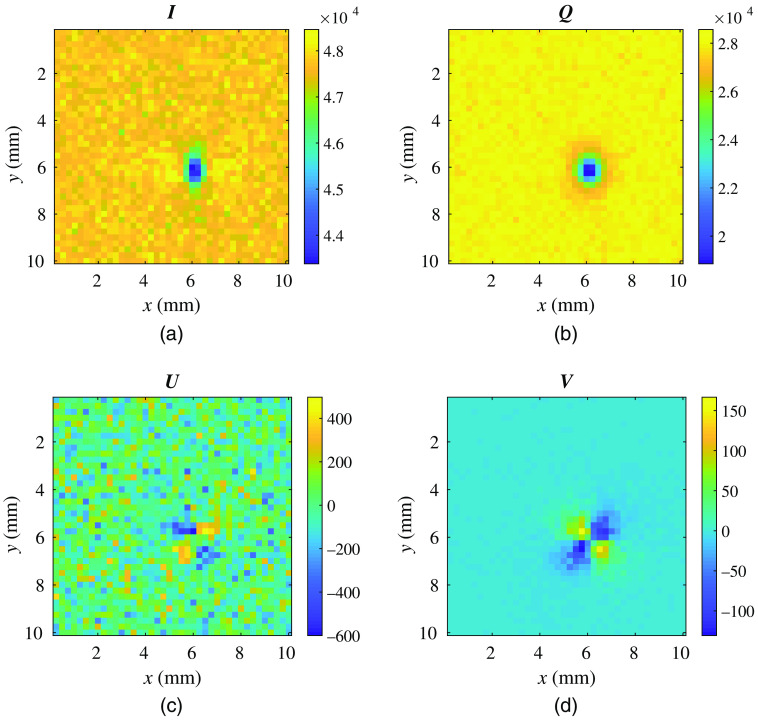
Distributions of (a) I, (b) Q, (c) U and (d) V backscattered from a $10\times 10\times 1.2\;{\mathrm{mm}}^{3}$ slab. A spherical inclusion of radius 0.5 mm is centered at (6,6,0.6) mm.

Finally, we show simulation of a slab-shaped medium embedded with a spherical inclusion, showcasing pMCX’s capability of modeling heterogeneous domains. In this benchmark (Fig. 4), the simulation domain is a $10\times 10\times 1.2\;{\mathrm{mm}}^{3}$ slab with a spherical inclusion of radius 0.5 mm centered at (6,6,0.6)  mm. The inclusion and the slab share identical absorption coefficient $\mu_{a}=0.005\;{\mathrm{mm}}^{-1}$, reduced scattering coefficient $\mu_{s}^{\prime }=1\;{\mathrm{mm}}^{-1}$, and refractive index n=1.33. However, the Mie scatters inside both domains are different. The background medium is filled with scatterers of radius r=0.05  μm and volume density $\rho =19.11\;\mu m^{-3}$; the inclusion is filled with scatterers of radius r=1  μm and volume density $\rho =1.11\times 10^{-3}\;\mu m^{-3}$. The choices of r and ρ values in either domain was computed based on the Mie theory to ensure their reduced scattering coefficients are the same. All spherical scatterings have a refractive index of, $n_{\mathrm{sph}}=1.59$. A $10\times 10\;{\mathrm{mm}}^{2}$ uniform planar light source is placed at the bottom (z=0  mm) surface, pointing toward the +z axis and emitting horizontally polarized light with the wavelength λ=632.8 nm. A cyclic boundary condition is applied to the four bounding box facets at ±x/±y directions to approximate an infinite slab and infinite-plane source. The incident Stokes vector is $\overset{\to }{S}=[1,1,0,0]$. A total of $2\times 10^{8}$ photons are simulated on an NVIDIA RTX 2080 GPU. We compare the distributions of backscattered [I,Q,U,V] at z=0 mm, as shown in Fig. 4. Because the inclusion and background slab share the same $\mu_{a}$, $\mu_{s}^{'}$, and n, a regular diffuse optics forward model without polarization capability would generate no contrast to the inclusion. However, our pMCX simulation has revealed distinct image contrasts in I, Q, and V images at the correct inclusion locations, suggesting the potential to detect tissue microstructure differences using polarized light. It is noteworthy that the noise level in each of the images is partially related to the anisotropy g determined based on the background scatterer parameters—a larger spherical radius results in a higher g value and less back-scattered photons. The significantly higher amplitude of inclusion contrast in Q image compared to that in I further demonstrates the advantages of using polarized imaging in detecting scattering differences compared to traditional diffuse optics where only I is typically measured.

## 4 Conclusion

In summary, we report a massively parallel implementation of polarized MC algorithm in our MCX simulator for modeling the propagation of polarized light inside complex media filled with spherical scatterers. Enabled by its built-in voxel-based geometric representation, the pMCX can handle arbitrarily heterogeneous media. We have described the preprocessing steps to encode the scattering properties of spherical particles with various radii and volume densities into a 3D voxel-based data structure. We provide validation and speed benchmarks ranging from simple homogeneous to complex heterogeneous domains. In all benchmarks, our pMCX solver reports excellent match with the widely used reference solver mcMeridian, while providing a speedup nearly three orders of magnitude. In addition, we observed different GPU memory utilization efficiency among global, constant, and shared memories, with the global memory implementation yielding the highest speed and least restriction. It is noteworthy that a limitation in both mcMeridian and pMCX simulations is that all media boundaries are assumed to have matched refractive indices. We plan to further extend this work to update the Stokes vector across mismatched boundaries.

## Code, Data, and Materials Availability

The pMCX algorithm has been incorporated in our widely disseminated MC simulator and is freely available at http://mcx.space/.
